# Chronic urticaria and COVID-19 vaccination: Qatar data (preliminary report of COVAC-CU-international)

**DOI:** 10.5339/qmj.2022.fqac.2

**Published:** 2022-04-04

**Authors:** Saleema Purayil, Sherin Thalappil, Maryam Al-Nesf, Emek Kocaturk

**Affiliations:** ^1^Allergy & Immunology Division Department of Medicine, Hamad Medical Corporation, Doha E-mail: SPurayil3@hamad.qa; ^2^Department of Dermatology, Koc University, Istanbul, Turkey

**Keywords:** allergic reaction, COVID-19 vaccine, CU

## Abstract

Background: It is a well-known fact that patients with chronic urticaria (CU) are not at a higher risk for a serious allergic reaction such as anaphylaxis from medications. However, there is a fear and some misconceptions regarding allergic reactions to the COVID-19 vaccine among patients and physicians, which might result in resistance to vaccination. Data about the incidence and severity of COVID-19 vaccine reactions in the CU population are scarce. In this study, we aimed to evaluate the real-world (Qatar) experience of the effects of COVID-19 vaccination on patients with CU and analyze the rates of vaccine-associated reactions and risk factors associated.

Methods: This is a cross-sectional questionnaire-based study conducted as a part of COVAC-CU international under the GALEN UCARE program. Adult patients with CU who received one or more doses of COVID-19 vaccination were administered a questionnaire regarding their demographic characteristics and any potential unfavorable effect of the vaccination from the November 03 to December 31, 2021.

Results: These are preliminary results from an ongoing study. The data were collected from 91 patients with CU, of whom 79.12% had chronic spontaneous urticaria, 15.3% had chronic inducible urticaria, and the remaining had both. Of these patients, 74.7% were women. The average age of the patients was 39.3 (range 15–68) years. The majority (84.6%) of them received 2 vaccine doses, 13.1% received 3 doses, and the remaining received 1 dose. Most (70.3%) of these patients did not experience any worsening in CU after vaccination. A total of 62.6% patients reported some type of side effects to the vaccine (16.4% had CU exacerbation and 46.1% other types of reactions, such as fever and muscle pain). None of the patients reported anaphylaxis. Two patients reported improvement in their symptoms.

Conclusion: Our local data suggest that patients with CU in Qatar can safely take the COVID-19 vaccine. Most patients with CU did not experience any worsening in symptoms, and there were no reports of a severe reaction (anaphylaxis). We recommend maximizing symptom control prior to vaccination to minimize the risk of worsening urticarial symptoms.

